# A compound-based proteomic approach discloses 15-ketoatractyligenin methyl ester as a new PPARγ partial agonist with anti-proliferative ability

**DOI:** 10.1038/srep41273

**Published:** 2017-01-24

**Authors:** Michele Vasaturo, Lorenzo Fiengo, Nunziatina De Tommasi, Lina Sabatino, Pamela Ziccardi, Vittorio Colantuoni, Maurizio Bruno, Carmen Cerchia, Ettore Novellino, Angelo Lupo, Antonio Lavecchia, Fabrizio Dal Piaz

**Affiliations:** 1 Department of Pharmacy, University of Salerno, Via Giovanni Paolo II, 132, 84084, Fisciano, Italy; 2 PhD Program in Drug Discovery and Development, University of Salerno, Via Giovanni Paolo II 132, I-84084 Fisciano SA, Italy; 3 Department of Sciences and Technologies, University of Sannio, Via Port’Arsa, 11, 82100 Benevento, Italy; 4 Department of Organic Chemistry, University of Palermo, Viale delle Scienze - Parco d’Orleans II, 90128, Palermo, Italy; 5 Department of Pharmacy, “Drug Discovery” Laboratory, University of Napoli “Federico II”, Via D. Montesano, 49, 80131 Napoli, Italy; 6 School of Medicine and Surgery, University of Salerno, via Salvatore Allende, 18, Baronissi, SA, Italy

## Abstract

Proteomics based approaches are emerging as useful tools to identify the targets of bioactive compounds and elucidate their molecular mechanisms of action. Here, we applied a chemical proteomic strategy to identify the peroxisome proliferator-activated receptor γ (PPARγ) as a molecular target of the pro-apoptotic agent 15-ketoatractyligenin methyl ester (compound **1**). We demonstrated that compound **1** interacts with PPARγ, forms a covalent bond with the thiol group of C285 and occupies the sub-pocket between helix H3 and the β-sheet of the ligand-binding domain (LBD) of the receptor by Surface Plasmon Resonance (SPR), mass spectrometry-based studies and docking experiments. **1** displayed partial agonism of PPARγ in cell-based transactivation assays and was found to inhibit the AKT pathway, as well as its downstream targets. Consistently, a selective PPARγ antagonist (GW9662) greatly reduced the anti-proliferative and pro-apoptotic effects of **1**, providing the molecular basis of its action. Collectively, we identified **1** as a novel PPARγ partial agonist and elucidated its mode of action, paving the way for therapeutic strategies aimed at tailoring novel PPARγ ligands with reduced undesired harmful side effects.

A crucial step in the search for and development of new drugs is the comprehensive understanding of the molecular mechanism of action of bioactive compounds. The mere scrutiny of the effects that chemical entities exert on cells, tissues or organisms is not enough to consider them for further uses or optimizations. Therefore, unbiased approaches aimed at the identification of the target(s) of promising molecules are emerging as a necessary starting point of many pharmaceutical and biochemical studies. In this field, proteomic-based strategies play a central role, as they potentially permit the identification of all possible interactors of a selected compound^1^.

In the present study, we used a chemical-proteomic approach based on compound-immobilized affinity chromatography^2^ to identify the protein target(s) of 15-ketoatractyligenin methyl ester (compound **1**, Fig. 1). This is a semi-synthetic ent-kaurane diterpene with interesting anti-proliferative and pro-apoptotic activities towards different cancer cell lines^3^, obtained through the inhibition of the PI3K pathway and thus of AKT^4^. The direct target of action of this compound is, however, still undefined.

Our proteomic results indicated peroxisome proliferator-activated receptor gamma (PPARγ) as a putative interactor of compound **1**. PPARγ is a member of the PPARs subfamily belonging to the nuclear receptors superfamily of ligand-inducible transcription factors^5^. It is a master gene of adipocyte differentiation and plays a key role in lipid and glucose metabolism and in the control of cell proliferation^6^. Consistently, several evidences indicate that PPARγ agonists induce apoptosis by inhibiting the PI3K/AKT pathway^7^,^8^,^9^,^10^. Hence, this protein is considered a pharmacological target of metabolic dysfunctions^11^ and neoplasias^12^. Thiazolidinediones (TZD) have been identified as PPARγ agonists and some of them have been approved for type 2 diabetes therapy^13^,^14^; however, concerns regarding their cardiovascular safety and possible hepatotoxicity have been reported^15^,^16^. Therefore, new PPARγ ligands are urgently required, possibly acting through a mechanism different from that of TZDs. Some bioactive compounds derived from plants have recently been described as promising PPARγ activators^17^. In order to validate proteomic-based data and to evaluate the effects of compound **1** on PPARγ activity, we used different analytic and bioanalytic techniques. Here, we provide experimental evidence on the binding mode and structural interactions of compound **1** with the PPARγ ligand binding domain (LBD), on its ability to act as a partial agonist and on the molecular basis of its PPARγ-dependent pro-apoptotic activity.

## Results

### Chemical proteomics identification of putative targets of compound 1

In order to identify possible molecular targets of compound **1**, we used a chemical proteomic approach, one of the most versatile methods to profile cellular targets of selected drug candidates based on compound-immobilized affinity chromatography^1^,^18^. To this goal, the hydroxyl group at position C-2 of compound **1**, not crucial to its biological activity^3^, was used to link the compound to an epoxy-activated sepharose resin (See Supplementary Figure S1). Reaction conditions were selected to prevent modification of the α,β-unsaturated carbonyl group shown to be essential for the activity of this class of compounds^19^,^20^. The obtained drug-linked beads were incubated with protein extracts from Jurkat cells (human T lymphoblast-like cell line), selected for their susceptibility to **1**, as previously reported^4^. After incubation for 30 minutes, the beads were extensively washed to remove non-specific interacting proteins and the tight-bound ones were eluted and digested using trypsin as proteolytic agent. Negative control experiments were simultaneously performed, using the same resin capped with ethanolamine. The obtained peptide mixtures were analysed by high-resolution nano LC-MS/MS, and the MS and MS/MS resulting data underwent bioinformatic elaboration by Mascot Search Engine software.

Chemical proteomic experiments were performed in triplicate and only those proteins identified in all the experiments were taken into account; proteins identified from both **1-**modified and control beads were excluded. Using this strategy, we identified the molecular chaperone heat shock protein 60 (Hsp60) and the nuclear receptor PPARγ as putative targets of **1** (Fig. 2a). These results were confirmed by Western blot analysis (See Supplementary Figure S2).

### Target validation by Surface Plasmon Resonance

Surface Plasmon Resonance (SPR) was employed to test the ability of compound **1** to interact with the proteins identified in the chemical proteomic experiments; to this goal, different concentrations of the diterpene were injected on the putative target proteins, singularly immobilized on sensor chips^21^. The obtained sensorgrams (Fig. 2b) showed an effective interaction of **1** with PPARγ; a software-aided elaboration of the results allowed measuring an equilibrium dissociation constant (K_D_) of 25.8 ± 5.5 nM for the **1**/PPARγ complex. SPR analysis was also performed using the immobilized PPARγ LBD to evaluate if this protein portion was involved in the interaction with **1**; the obtained results (K_D_ of 39.2 ± 3.9 nM) were almost superimposable with those achieved using the whole protein, indicating that **1** binds that protein region. Conversely, no affinity of the diterpene towards Hsp60 was observed (Fig. 2c).

**Figure 1:**
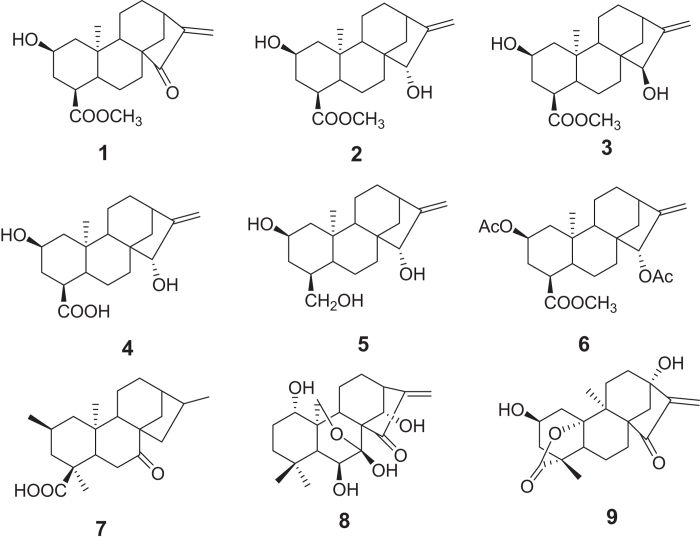
Structures of compounds 1–9.

To assess the potential of **1** to act as a PPARγ modulator, we investigated the structural and functional aspects of its interaction with this nuclear receptor. To define the structural features required by **1** for an efficient interaction with PPARγ, as a first step, we performed SPR analyses using some diterpenes displaying structural relationship with **1** (compounds **2–9**, Fig. 1). Rosiglitazone, a well-characterized PPARγ full agonist^22^, was used as positive control. All these compounds displayed an affinity towards PPARγ lower than that of **1** (Table 1 and Supplementary Figure S3).

In particular, the lack of the α,β-unsaturated keto group significantly affected the interaction with PPARγ, strongly increasing both thermodynamic (K_D_) and kinetic (k_off_) dissociation constants (cfr. compounds **2** and **3** *vs* **1**). Compounds **2** and **3** share the same scaffold and type of substituents on ring A, lacking only the α,β-unsaturated carbonyl moiety in comparison to **1**. Since this modification results in a decreased ligand affinity for PPARγ, it is tempting to speculate that these compounds do not covalently bind to but can still weakly interact with PPARγ. Diterpenes **4**–**7** with different substitution pattern on rings A-B are completely inactive, suggesting a critical role of hydroxyl group in 2 and methyl ester in 4 for binding to the receptor. In contrast, compounds **8** and **9**, even if contain the α,β-unsaturated carbonyl moiety, adopt a substantially different conformation of the perhydrophenanthrene nucleus, leading to severe steric clashes with the protein. This might impair the access of the ligands to the PPARγ LBD, explaining why their binding is not detected in SPR analysis.

**Figure 2:**
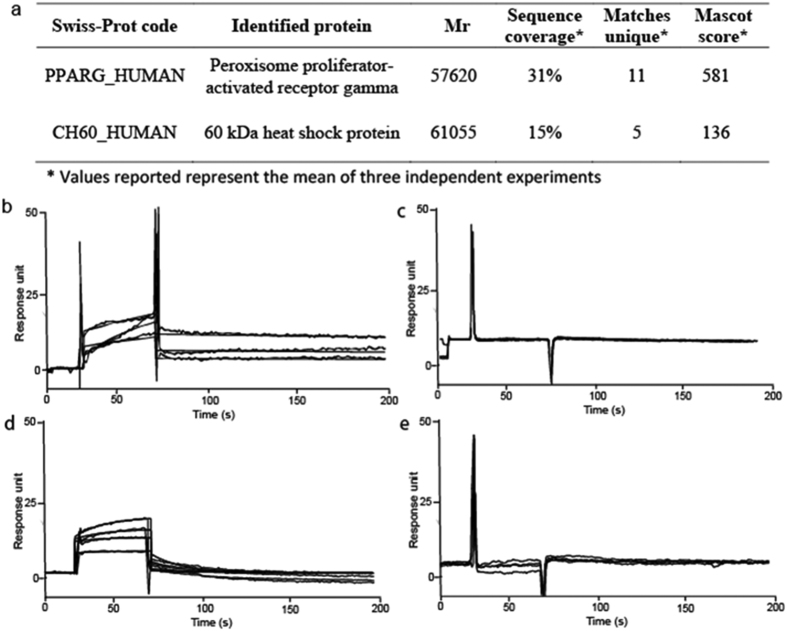
Identification and validation of 1 putative targets. (**a**) Proteins identified by chemical proteomic experiments as putative **1** molecular targets. SPR sensorgrams achieved injecting different concentrations (from 0.025 to 1 μM) of compound **1** on immobilized PPARγ (**b**), Hsp60 (**c**), PPARα (**d**) and PPARδ (**e**).

In order to evaluate if **1** acts as a selective ligand of PPARγ, SPR analyses were also carried out on immobilized PPARα and PPARδ, two proteins structurally and functionally related to PPARγ^23^,^24^. No binding occurred between **1** and PPARδ; conversely, some interaction with PPARα was observed, the measured K_D_ (1.32 ± 0.08 μM) for **1/**PPARα complex was about 50-fold higher than that detected for the **1**/PPARγ complex (Fig. 2d,e). Comparison between the sensorgrams achieved for **1**/PPARα and **1**/PPARγ interactions revealed that the binding phases were similar, but the dissociation kinetics were clearly different: the **1**/PPARα complex was completely dissociated after less than 50 s, whereas the **1**/PPARγ complex dissociation required longer times and was incomplete.

### Compound 1 covalently binds to PPARγ C285 residue

**Figure 3:**
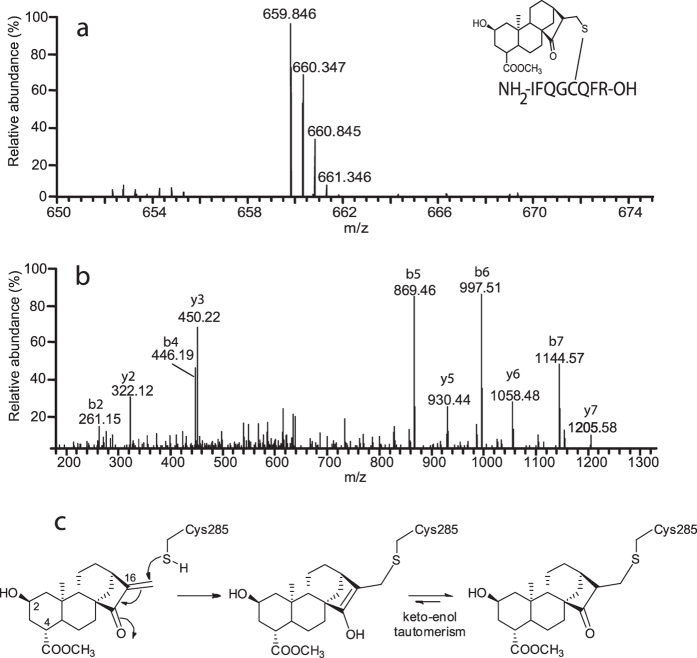
Compound 1 interaction with PPARγ is stabilized by the formation of a covalent bond. (**a**) High-resolution mass spectrum and (**b**) MS/MS data of peptide 281–288 covalently bound to **1**. Peptide sequence is also reported. (**c**) The covalent coupling of the ligand to C285 of PPARγ is the result of a Michael addition.

The high stability of the **1**/PPARγ complex and the presence in the PPARγ-LBD sequence of a cysteine residue (C285) highly reactive towards nucleophilic groups such as α,β-unsaturated ketones^25^, prompted us to investigate whether **1** could form a covalent bond with the protein. To this purpose, we investigated the presence of covalently modified peptides by performing a classic MS-based peptide mapping on the **1**/PPARγ-LBD complex using trypsin as proteolytic agent. This analysis covered most of the protein sequence (See Supplementary Table S1); besides, a doubly charged ion at *m*/*z* 665,844 was observed, suggesting that **1** was covalently bound to peptide 281–288 (Fig. 3a). This hypothesis was confirmed by MS/MS analysis of this ion (Fig. 3b) that revealed the modification at C285 via Michael addition (Fig. 3c).

To verify whether compound **1** could form a covalent bond with Hsp60, we performed the same protocol as above. Also in this case, the analysis covered most of the protein sequence and detected all the cysteine-containing peptides, but no peptide covalently modified by compound **1** (See Supplementary Table S2). The result of this experiment and the analysis of the SPR data indicated that Hsp60 cannot be considered a target of compound **1**; its occurrence as a “false positive” is not very surprising as Hsp60 isoforms have often been identified in proteomic-based protein interaction studies (197 times on 411 studies on human cells reported on CRAPome database – http://www.crapome.org/), whereas PPARγ was not identified in any of them.

To further confirm that compound **1** forms a covalent bond with PPARγ, we performed the SPR experiments as described, with the protein immobilized on the sensor chip after a 2 h incubation with a 5-fold molar excess of **1**. As expected, this pre-incubated protein did not interact with injected **1** (See Supplementary Figure S4).

### Structural characterization of the 1/PPARγ complex

We employed the covalent docking protocol CovDock^26^,^27^ implemented in the Schrödinger Suite to visualize possible conformational states of the C285 thioether resulting from the reaction with **1**. CovDock uses different tools of the Schrödinger Suite to mimic distinct stages of covalent inhibitor binding. The first step is a classical non-covalent docking with an alanine mutation of the nucleophilic side chain followed by an automated bond formation and a second docking step with the covalent bond in place. The basic concept of the software is that a covalently bound ligand has to adopt an energetically favorable unbound pose before bond formation occurs and that these unbound poses do not dramatically change during the reaction pathway because conformational sampling is done solely prior the non-covalent docking step.

The large number of crystal structures published in the PDB and the induced-fits in the PPARγ binding pockets upon ligand binding made difficult the choice of a crystal structure for docking. For instance, residues F282, R288, F363 and Y473 adopt different conformations among the available crystallographic structures and induce alterations in volume and shape of the binding site, allowing a better binding of the ligand^28^,^29^,^30^. Moreover, the region between H2′ and H3 of PPARγ, called Ω-loop, comprises the most flexible part of the LBD and has recently been suggested to be an important modulator of PPAR function in addition to or in combination with H12^31^,^32^,^33^. This region is usually poorly structured in many PDB structures, while in others it is ordered. For these reasons, we adopted a systematic PDB selection process in order to assess which crystal structure should be used as a model to accurately dock compound **1** into the protein. Virtual screening was conducted on a number of PPARγ crystal structures using a library of known PPARγ partial agonists and a set of decoy compounds. Each docking model was evaluated using receiver operating characteristic (ROC) curves and enrichment factors. The best performing crystal structure for docking was found to be 3B3K^34^ with a ROC value of 0.80 and an enrichment factor of 9.5 at 2% of the virtual screening. Details about the PDB selection process are reported in the Experimental Procedures section.

**Figure 4:**
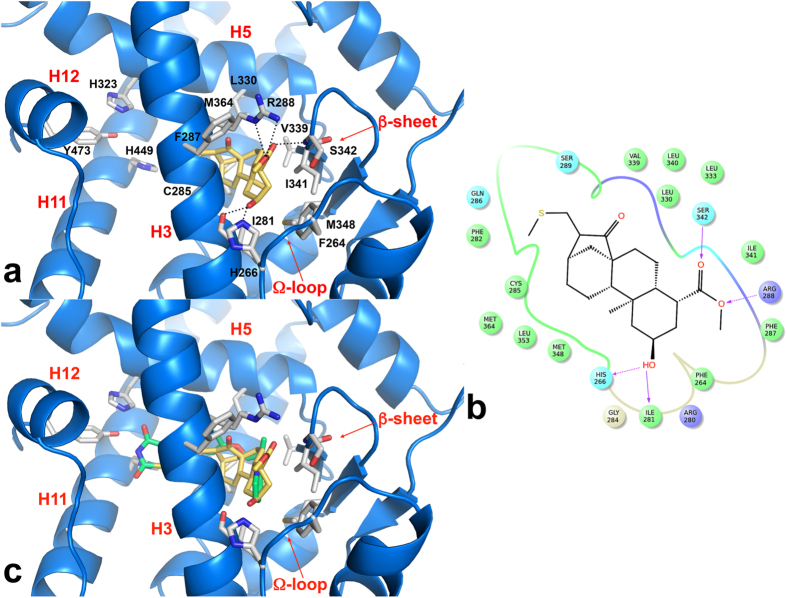
*In silico* docking of compound 1 into the PPARγ binding pocket. (**a**) Binding mode of compound **1** (yellow sticks) into the PPARγ binding site represented as a blue marine ribbon model. Only amino acids located within 4 Å of the bound ligand are displayed (white sticks) and labelled. The Ω-loop, a flexible loop region between H2′ and H3, and the β sheet region of the LBD, are displayed. H-bonds discussed in the text are depicted as dashed black lines. The 2D ligand-interaction diagram of **1** is depicted in panel (**b**). Positively charged amino acids are represented with dark blue circles, polar amino acids are represented with light blue circles and hydrophobic amino acids are represented with green circles. Hydrogen bonds are depicted with purple arrows–dashed arrows for H-bonds involving amino acid side chain and regular arrows for H-bonds involving amino acid backbone. (**c**) C^α^ superposition of the complexes of PPARγ with compound **1** and Rosiglitazone (green sticks, PDB code 2PRG).

The PPARγ ligand-binding pocket has a Y-shaped form and can be divided into two sub-pockets, a) the activation function 2 (AF-2) and b) the β-sheet sub-pocket^35^. In order to activate PPARγ, partial agonists bind only the β-sheet sub-pocket, while full agonists always occupy both AF-2 and β-sheet sub-pockets^36^. A low-energy pose of **1** covalently bound to C285 was predicted by CovDock and this conformation, which adopts a *R* configuration at position 16 of the Michael acceptor, is stabilized by several H-bonds with the key residues in the β-sheet sub-pocket. Unlike Rosiglitazone, which takes a U-shape conformation in the ligand-binding pocket and wraps around H3 to directly contact the AF-2 helix (H12), compound **1** occupies the region delimited by H3 and β-sheet (β-sheet sub-pocket) and makes no contact with H12 or residues involved in co-activator recruitment (Fig. 4a,c), as already observed in structures of complexes with partial agonists, such as those with BVT.13, MRL-24 and nTZDpa^37^. In contrast, the full agonist Rosiglitazone occupies roughly 40% of the PPARγ ligand-binding site in a U-shaped conformation and consists of a polar head and hydrophobic tail. The polar head makes a net of H-bonds with S289, H323, H449 and Y473 side chains, while forming a hydrophobic region with F363, Q286, F282 and L469. Despite the fact that **1** binds in a different mode from Rosiglitazone, occupying the β-sheet sub-pocket, the *ent*-kaurane skeleton of **1** overlaps the hydrophobic region of Rosiglitazone when the two structures are superposed (Fig. 4b).

The carbonyl and the ethereal oxygen atoms of the ester group at position 4 of the ligand form two H-bonds, one with the NH backbone of S342 (located at the β-sheet) and one with both NH_2_ and N^ε^ of R288 side chain on H3. The hydroxyl group at position 2 establishes a H-bond with the C=O backbone of I281 (d_OH-O_ = 2.8 Å) as well as a very weak H-bond (d_OH-N_ = 4.1 Å) with the side chain of H266, located at the Ω loop that links H2 to H3. Non-polar contacts are observed along the full extension of the **1** molecule. These contacts start at the Ω-loop of the protein, and extend all the way through the ligand-binding pocket. Residues involved in these interactions include F264 (part of the Ω-loop), I281, G284, and F287 on H3, V339, I341 and M348 on the β-sheet, L330 (H5), and M364 (H7). It is important to note that the Ω-loop is highly unstable and the residues within this loop are quite flexible; thus, the observed ligand/Ω-loop contacts cannot be easily quantified and must be interpreted accordingly.

### Compound 1 is a PPARγ activator and requires the binding to C285 for its transactivation potential

**Figure 5:**
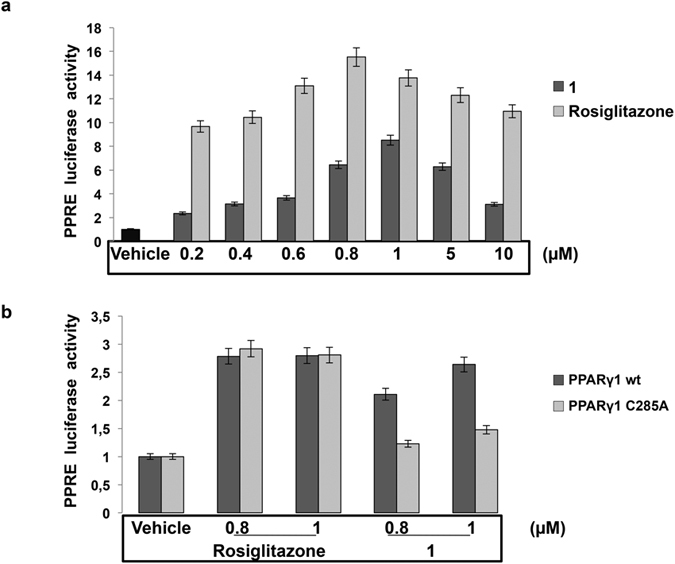
PPARγ transactivation activity of compound 1. (**a**) Human HEK293 cells stably expressing an exogenous Flag-tagged wild type PPARγ1 were transiently transfected with the PPRE-luciferase reporter gene and treated for 24 h with Rosiglitazone or compound **1**, respectively, at the indicated doses. Luciferase activity is reported as fold-induction after normalization to β-galactosidase activity used as control for transfection efficiency. The results are further normalized to those from cells treated with vehicle only (e. g. DMSO in the absence of compound **1** or Rosiglitazone). Data are the mean ± SD of three independent experiments performed in duplicate. (**b**) Human HEK293T cells were co-transfected with the PPRE-luciferase reporter gene and the wild-type PPARγ1 or its mutant version (C285A). After transfection, cells were treated for 24 h with Rosiglitazone or **1** at the indicated concentrations. Luciferase activity is reported as fold-induction after normalization to β-galactosidase activity used as control for transfection efficiency. The results are further normalized to those from cells treated with vehicle only (e.g. DMSO in the absence of compound **1** or Rosiglitazone). Data are the mean ± SD of three independent experiments performed in duplicate.

To verify that **1** is a *bona fide* PPARγ ligand with transactivation ability, we transiently transfected a PPRE-TK-luciferase-reporter plasmid in HEK293 cells that, in addition to the endogenous protein, stably express an exogenous PPARγ. These cells were selected as they express a fixed and known amount of PPARγ, so that the differences in luciferase activity can be ascribed to the different ligand used. Cells were treated with increasing concentrations of Rosiglitazone or **1** for 24 h and luciferase activity evaluated. Rosiglitazone induced luciferase activity in a dose-dependent manner reaching a peak at 0.8 μM and declining at higher concentrations (Fig. 5a). Compound **1** displayed a transactivation activity that was about 40% lower than Rosiglitazone, with a peak at 1 μM.

At relevant concentrations, many small molecules self-associate into colloidal aggregates that act nonspecifically on protein targets and exhibit bell-shaped concentration–response curves^38^. To mitigate this, use of nonionic detergents, which can disrupt aggregates, is now common in target-based screens^39^. We then checked whether addition of a detergent, such as 0.001% Tween 20, to the medium could affect the aggregation status of the ligand and hence the transactivation effect on the luciferase reporter gene. We did not find any variation in the bell-shaped activity profile of compound **1** (Supplementary Figure S5). So, we assume that the reduced reporter gene transcription efficiency operated by **1** is independent of the status of the ligand. All together, these results clearly demonstrate that **1** behaves as a partial agonist of PPARγ.

To confirm that the activity of **1** requires the formation of a covalent bond with C285, we tested the ability of this compound to transactivate a PPARγ mutant in which the cysteine residue at position 285 was replaced by an alanine residue. To this goal we transiently transfected basal HEK293T cells with the PPRE-TK-luciferase-reporter plasmid along with the expression vector for the wild-type PPARγ1 or the mutant version carrying the C285A substitution^40^. Cells were exposed either to the vehicle alone or to the two concentrations of Rosiglitazone and **1** that displayed the highest induction for 24 h and luciferase activity measured in the cell extracts after 24 h further. Rosiglitazone stimulated equivalent luciferase activity with both the wild type and mutant receptor. Compound **1**, in contrast, stimulated 40% less luciferase activity than Rosiglitazone with the wild type receptor, in line with being a partial agonist; strikingly, it induced even lower luciferase activity with the C285A mutant (Fig. 5b). These results indicate that the C285A mutation does not influence Rosiglitazone-induced PPARγ transactivation activity, while it significantly impairs that elicited by **1**, demonstrating that the binding to this amino-acid residue is absolutely required for PPARγ-induced transactivation.

### Compound 1 inhibits the growth of HT-29 colon-cancer derived cell lines

**Figure 6:**
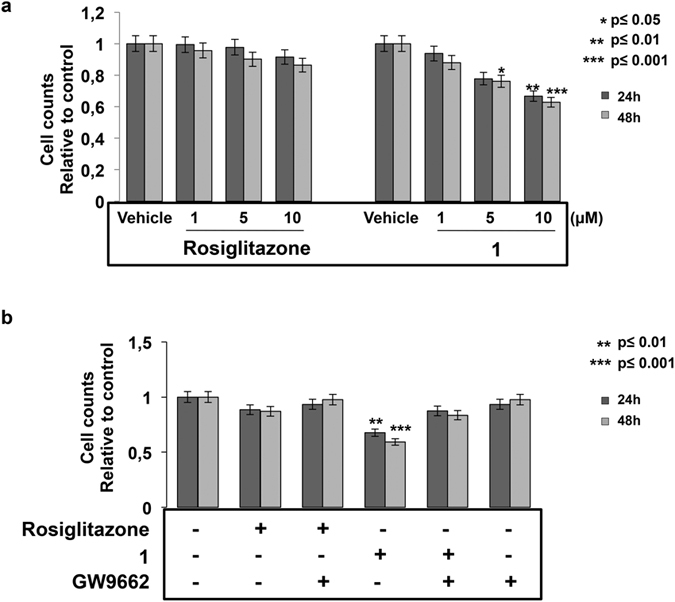
Compound 1 inhibits cell growth in a dose- and time-dependent manner. (**a**) Exponentially growing HT-29 cells were treated with increasing doses of Rosiglitazone or **1** (1, 5 and 10 μM) for 24 and 48 h, harvested and counted. Data are the mean ± SD of three independent experiments performed in duplicate giving similar results. Significance is indicated as *p ≤ 0.05, **p ≤ 0.01 and ***p ≤ 0.001. (**b**) HT29 cells were seeded in 24-well in order to reach the density of cells/cm^2^ in standard conditions (10^5^ cells). After 24 h, cells were treated with GW9662 (10 μM) for six hours, exposed to Rosiglitazone or **1** (10 μM) for 24 and 48 h, collected and counted. The experiments were performed in triplicate and the data expressed as mean ± SD. Significance is indicated as **p ≤ 0.01 and ***p ≤ 0.001.

It has been reported that compound **1** affects the PI3K/AKT pathway and hence cell growth and proliferation while it induces apoptosis^4^. Based on the achieved results on the binding and transactivation potential of **1** towards PPARγ, we tested whether these effects were strictly dependent upon the binding to this receptor. HT-29 colon-cancer derived cells were cultured for 24 and 48 h in the presence of increasing amounts of **1** or Rosiglitazone and the number of surviving cells evaluated by automatic cell counter (Fig. 6a).

Compound **1** displayed a more pronounced dosage-dependent inhibition of cell growth than Rosiglitazone. To assess whether this anti-proliferative effect could be ascribed to the direct binding to PPARγ, we exposed HT-29 cells to GW9662, an irreversible PPARγ antagonist, for 6 h and, subsequently, to **1** for additional 24 and 48 h. The sequential treatment with both molecules (GW9662 and **1**) resulted in increased cell counts with respect to cells exposed to **1** alone, suggesting that inhibition of cell proliferation is mediated by PPARγ activation (Fig. 6b). A less pronounced effect was observed upon Rosiglitazone treatment.

**Figure 7:**
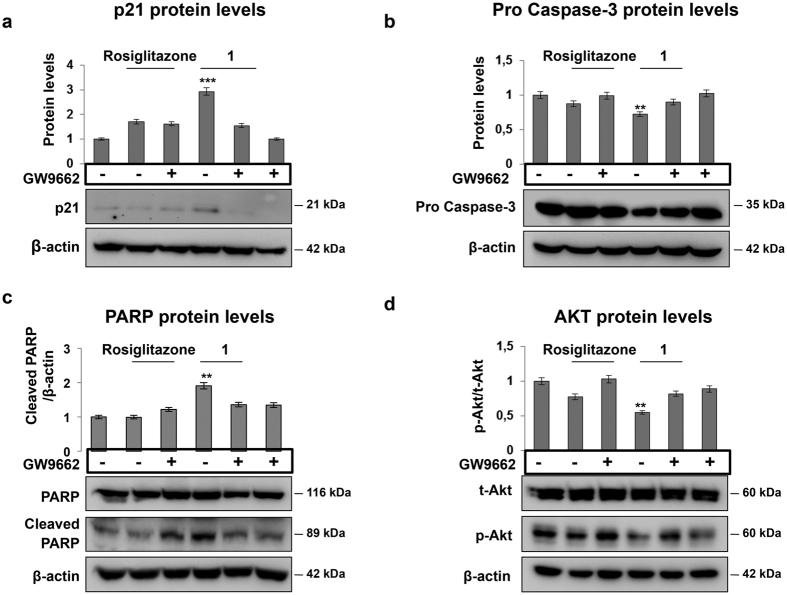
Anti-proliferative and pro-apoptotic activities of compound 1 are dependent on PPARγ activation. Western blotting analysis performed on total protein extracts from HT29 cells pretreated with GW9662 for 6 h and exposed to Rosiglitazone or compound **1** for 6 h to detect p21^waf1/cip1^ (**a**) AKT (**d**) and for 48 h to assess Caspase-3 (**b**) and PARP (**c**) expression. β-actin was used for normalization of the loaded samples. The obtained results were further normalized as follows: control cells exposed to DMSO as vehicle (lane/column 1) or to GW9662 (lane/column 6); cells exposed to Rosiglitazone alone (lane/column 2) or after pretreatment with GW9662 (lane/column 3); cells exposed to compound **1** alone (lane/column 4) or after treatment with GW9662 (lane/column 5). The graphs in (**a**–**c**) represent the mean ± SD of the protein/β-actin ratio from two independent experiments for p21^waf1/cip1^, Caspase-3 precursor and cleaved PARP proteins, respectively. The graph in (**d**) represents the mean ± SD of pAKT/AKT ratio from two independent experiments. Significance is indicated as **p ≤ 0.01 and ***p ≤ 0.001.

To further support these results, we assessed the expression of some well-known markers of cell proliferation and apoptosis: p21^waf1/cip1^ levels sharply increased upon exposure to **1** alone, while they diminished after pre-treatment with GW9662 (Fig. 7a). In addition, the expression of two components of the apoptotic pathway, such as caspase 3 and its substrate Poly (ADP-ribose) polymerase (PARP), was affected resulting in a reduction of the pro-caspase 3 precursor and an increase of the PARP cleaved form. Pre-treatment with GW9662 counteracted both the effects (Fig. 7b,c). We then assessed the effect of GW9662 on the levels of the phosphorylated form of AKT (p-AKT) to prove that **1** inhibits cell proliferation *via* the PI3K/AKT pathway^4^. Treatment with **1** alone caused a robust reduction of p-AKT, while pre-treatment with GW9662 resulted in its persistence, as shown by the western blot experiments of Fig. 7d. Altogether these results demonstrate that the anti-proliferative and pro-apoptotic effects of **1** are mediated by the binding to PPARγ.

## Discussion

The list of biologically active natural and synthetic compounds acting as selective ligands of nuclear receptors (NRs) LBD is increasing every day. NRs are typically located in the nucleus, bound to the DNA mostly as heterodimers with the retinoid X receptor (RXR). In the absence of ligands, they are complexed with co-repressor proteins; ligand binding causes dissociation of the co-repressors and recruitment of co-activators. The NR-DNA complex establishes additional contacts with the basal transcriptional machinery, including RNA polymerase II, in order to start the transcription process. The molecular mechanism that regulates the alternative interactions of NRs with co-activators or co-repressors has been decoded by crystallographic studies^41^.

The intensity and the quality of the ensuing biological response depend on the LBD conformational changes dictated by the differential binding potential that can either strongly (full agonists) or weakly (partial agonists) stimulate or, alternatively, inhibit (antagonists) gene transcription^42^. As members of the NR superfamily, also PPARs act as ligand-dependent transcription factors and regulate the expression of genes involved in lipid metabolism, adipocyte differentiation, glucose metabolism and insulin sensitivity, inhibition of cancer cell proliferation and inflammation^43^,^44^,^45^. Specifically, canonical full agonists interact with the PPARγ-LBD, stabilize the AF2 transactivation domain on H12 and, consequently, expose the interacting surface necessary for the interaction with co-activators and components of the transcriptional machinery^44^.

In the present report, by using different methodologies we demonstrate that **1** is a selective PPARγ ligand. The binding appears to be highly selective for PPARγ as SPR comparative affinity studies show no significant binding with PPARδ and only a weak interaction with PPARα. In the docking model, **1** is positioned far from H12 and binds close to, and strongly stabilizes, the H3/β-sheet/Ω-loop region of the LBD, similar to other PPARγ partial agonists such as BVT.13, MRL-24 and nTZDpa^37^. We recently demonstrated that also cladosporol B, a secondary metabolite from *Cladosporium tenuissimum*, makes no direct contacts with H12 residues, a hallmark of full agonists such as TZDs, but preferentially stabilizes H3 through closer hydrophobic contacts or H-bonds made with residues of this helix (S289, F282, Q283 and Q286)^46^ (Fig. 4). Of note, these residues are the same involved in the binding of **1** to PPARγ, suggesting that PPARγ partial agonists follow a common mode of binding to the receptor through H3 while H12 adopts a highly dynamic conformation. Previous studies suggested that fatty acid metabolites activate PPARγ through structural rearrangements of the Ω-loop and distinct degrees of the receptor mediated cellular activities originate from structural differences in this region^33^. On the basis of this reasoning, it is conceivable to argue that the activity of partial agonists may stem from a mechanism distinct from that of full agonists with H3, β-sheet and the Ω-loop directly involved in this modality^37^,^40^,^47^,^48^,^49^. Interestingly, and at odds with other partial agonists, **1** covalently binds to the C285 present in the PPARγ-LBD through a α,β-unsaturated ketone. Here we clearly demonstrate that the binding to PPARγ is strictly dependent on this cysteine residue at position 285, as a site-directed mutation impairs the transactivation ability (Figs 3 and 5). This residue has been shown to be essential for the activity and covalent binding of some PPARγ agonists such as 15d-PGJ_2_^25^, indicating that the same amino acid residue may be crucial to the binding of both full and partial agonists^50^,^51^. Further investigations are necessary to clarify this aspect. Notably, the C285 residue is conserved in the three types of human PPARs as well as in their orthologs in other species, but not in other nuclear receptors, suggesting that the covalent bond formation could be a feature for all PPAR isoforms. The data reported here, however, suggest that C285 may have a different role for full or partial agonists. On the other hand, it is important to underline that the presence of a α,β-unsaturated ketone is not enough to achieve PPARγ agonism, as inferred by the results obtained on compounds **8** and **9**. Finally, the identification of PPARγ as a target of **1** provided the molecular basis of the previously reported anti-proliferative and pro-apoptotic effects of **1**^4^. We demonstrate that these activities are mediated through inhibition of the PI3K/AKT pathway, in accordance with *in vitro* data obtained using other known PPARγ activators^7^,^8^,^9^,^10^. By using the irreversible PPARγ antagonist GW9662, we reinstate HT-29 cells ability to proliferate, counteracting the inhibition of the PI3K/AKT pathway (Figs 6 and 7). On the basis of these results, we believe that **1** could be a promising lead for the development of new therapeutic and/or biochemical tools endowed with both anti-proliferative and pro-apoptotic as well as anti-diabetic properties.

## Experimental Procedures

### Reagents

Solvents and water (HPLC grade) were purchased from Romil (ROMIL Ltd, Cambridge, UK). Recombinant human PPARγ-LBD, consisting of the region 195–477 of PPARγ, was from Bertin-Pharma (Bertin-Pharma, Montigny le Bretonneux, France); recombinant human PPARα, PPARγ, PPARδ and Hsp60 were acquired from Tebu-Bio (Tebu-Bio, Megenta, Italy). Rosiglitazone, GW9662, d-luciferin sodium salt, trichloroacetic acid, propidium iodide (PI) were from Sigma Aldricht (St. Louis, MO, USA). Compounds **1**–**7** were synthesized as reported elsewhere^3^; compounds **8** and **9** were selected from those present in the natural compound library of the Department of Pharmacy of the University of Salerno. Purification and structural characterization of these compounds were previously published^18^,^52^.

### Cells and antibodies

Jurkat, HT-29 and HEK293 cells were obtained from the American Type Culture Collection (Rockville, MD, USA). Jurkat cells were cultured as described in Ref 4 while HT-29 and HEK293 cells as reported in Ref 44. Antibodies against p21^waf1/cip1^, β-actin and caspase 3 precursor were purchased from Santa Cruz Biotechnology (Santa Cruz, CA, USA); AKT, p-AKT and PARP from Cell Signaling (Danvers, MA, USA), anti-PPARγ (ab59256) and anti-Hsp60 (ab46798) from Abcam (Cambridge, United Kingdom); anti-mouse and anti-rabbit IgG peroxidase-linked secondary antibodies, ECL and ECL Plus Western blotting detection kit from Amersham Life Science (Little Chalfont, Buckinghamshire, UK). Dulbecco’s Modified Eagle’s Medium (D-MEM), FBS, penicillin-streptomycin, l-glutamine, trypsin-EDTA and OptiMEM I were from Gibco (Carlsbad, CA, USA), charcoal/dextran-treated FBS was from Hyclone (Logan, Utah, USA).

### Chemical proteomics

One milligram of **1** was incubated at 30 °C with 8.5 mg of an epoxy-activated sepharose resin 6B (Sigma-Aldrich) in 500 μl of 30 mM NaHCO_3_, 40% (v/v) CH_3_CN (pH 8), to achieve compound immobilization. The reaction was monitored by LC/MS using a LC-Q Advantage instrument coupled with an Accela HPLC system (Thermo Fisher Scientific, Waltham, MA, USA) and was completed after 4 h, leading to a compound **1** concentration of about 10 mmol for 1 ml of resin. Un-reacted resin epoxy groups were deactivated by adding 50 μl of 1 M ethanolamine. Control resin was prepared directly incubating 8.5 mg of an epoxy-activated sepharose resin 6B with 1 M ethanolamine.

For protein extracts, control or treated cells were harvested by using a solution of *trypsin*-EDTA and washed three times with phosphate buffer saline (10 mM NaH_2_PO_4_, 137 mMNaCl, 2.7 mMKCl pH 7.4, PBS). Cells were collected by centrifugation for 10 min at 400 g and lysed for 30 min on ice in PBS containing 0.1% Igepal (lysis buffer) and a protease and phosphatase inhibitor cocktail (P8340, Sigma-Aldrich). Samples were clarified by centrifugation for 15 min at 15000 g at 4 °C. Protein concentration was determined by Bio-Rad DC Protein Assay (Bio-Rad, Hercules, CA, USA) using bovine serum albumin as a standard.

Jurkat cell lysates (500 μg) were incubated with **1**-loaded or with control resin for 2h at 25 °C. The beads were washed three times with the lysis buffer and then three times with PBS. Interacting proteins were eluted by 50 μl of Laemmli buffer (60 mMTrisHCl pH 6.8, 2% sodium dodecylsulfate, 10% glycerol, 0.01% blue bromophenol, 5% β-mercaptoethanol). Eluted proteins were separated on a 12% SDS-PAGE and stained with Brilliant Blue G-Colloidal (Sigma-Aldrich). Peptides were analysed as reported elsewhere^18^ by high resolution LC-MS/MS, using an Orbitrap XL mass spectrometer (Thermo Fisher Scientific Inc., Rockford, IL USA) equipped by a nanospray ion source and coupled with a nano-Acquity capillary UPLC system (Waters, Milford, MA, USA).

### Surface plasmon resonance (SPR)

SPR analyses were carried out on a BIACORE 3000 instrument (GE-Healthcare). PPARα, PPARγ, PPARδ and HSP60 surfaces were prepared on research−grade CM5 sensor chips (GE Healthcare). Proteins (100 μg/ml in 10 mM CH_3_COONa, pH 5.0) were immobilized using a standard amine−coupling protocol, to obtain densities of 3–5 kRU. Testing compounds were dissolved in 100% DMSO to obtain 4 mM solutions, and then diluted in PBS containing variable amounts of DMSO, achieving a final DMSO concentration of 0.1%. For each molecule a five−point concentration series (25 nM, 65 nM, 150 nM, 400 nM, 1 μM) was set up. SPR experiments and data elaboration were carried out as reported elsewhere^53^.

### PPARγ-LBD and Hsp60 peptide mapping

PPARγ-LBD and Hsp60 were incubated with a 2:1 molar excess of **1** under stirring for 15 min in PBS at 37 °C. Eluted proteins were loaded on a mono-dimensional 12% SDS-PAGE, stained with Brilliant Blue G-Colloidal, reduced, and digested by trypsin. The resulting fragments were extracted and analyzed by LC-MS/MS using the same instrument and protocol described before. Peptide identification was performed by MS and MS/MS data using Mascot (Matrix Science) to interrogate the Swiss Prot non-redundant protein database. Settings were as follows: mass accuracy window for parent ion, 10 ppm; mass accuracy window for fragment ions, 200 millimass units; fixed modification, carbamidomethylation of cysteines; variable modifications, oxidation of methionine and compound **1** addition (+330, 1831) as custom modification.

### Computational chemistry

Molecular modelling and graphics manipulations were performed using Maestro [Maestro, version 10.1, Schrödinger, LLC, New York, NY, 2015] and UCSF-Chimera 1.8.1 software packages^54^ running on a E4 Computer Engineering E1080 workstation provided of a Intel Core i7-930 Quad-Core processor. CovDock algorithm^26^ of the Schrodinger Small Molecule Drug Discovery Suite was used for all docking calculations. Figures were generated using Pymol 1.0.

### PPARγ structure selection

Several crystal structures of PPARγ in complex with partial agonists are available in PDB. To reduce the list size and implement a systematic PDB selection process, several exclusion criteria were applied to the structures: i) only structures with cocrystallized drug-like compounds were chosen; ii) no mutated protein structures were included; iii) repeated ligand-protein complexes were excluded. Moreover, only cocrystallized ligands characterized by high potency (EC_50_ ≥ 10 μM) and able to activate the receptor from 15% up to a maximum of 80%, compared to the full agonist control in a transactivation assay, were included. We thus obtained 31 entries; a superposition of all these structures on the alpha carbon atoms showed that several of them lack the Ω-loop, a flexible region between H2′ and H3 which was reported to play an important role in binding of PPARγ ligands^31^,^32^,^33^. Thus, we selected the PPARγ structures in which the Ω-loop was fully solved and collected a total of six structures: 2I4P (resolution: 2.1 Å)^55^, 2I4Z (resolution: 2.25 Å)^55^, 3B3K (resolution: 2.6 Å)^34^, 2G0G (resolution: 2.54 Å)^28^, 2G0H (resolution: 2.3 Å)^28^, and 4PRG (resolution: 2.9 Å)^56^.

### Protein preparation

The six selected PPARγ crystal structures were processed through the Protein Preparation Wizard in Maestro^57^. The right bond orders as well as charges and atom types were assigned and the hydrogen atoms were added to protein. Arginine and lysine side chains were considered as cationic at the guanidine and ammonium groups, and the aspartic and glutamic residues were considered as anionic at the carboxylate groups. All crystallographic water molecules were deleted. Imidazole rings of H449 and H323 into PPARγ were set in their N^ε^ 2-H (N *tau*-H) tautomeric state. Moreover, an exhaustive sampling of the orientations of groups, whose H-bonding network needs to be optimized, was performed. Finally, the protein structures were refined with a restrained minimization with the OPLS2005 force field^58^ by imposing a 0.3 Å root-mean-square deviation (rmsd) limit as the constraint.

### Ligand preparation

A set of 30 known PPARγ partial agonists (Supplementary Figure S6) was selected from the literature and built using the chemical editor included in Maestro software. LigPrep was used to generate multiple tautomers and/or protonation states for each compound at the pH range of 7.0 ± 2.0; structures were then minimized using the OPLS_2005 force field.

A set of 1000 drug-like decoy compounds with an average molecular weight of 400 g/mol was downloaded as a 3D SD file from the Schrödinger website (http://www.schrodinger.com)^59^,^60^. Although the affinity of the decoy compounds for PPARγ is unknown, the decoys are structurally dissimilar to the 30 active PPARγ partial agonists. As such, the decoys do not possess the common structural features required for binding to PPARγ and therefore are unlikely to be active themselves. The drug-like decoy set was prepared in the same way as the active compounds, including generation of multiple tautomeric forms and ionization states.

### Cognate ligand docking

Pose generation quality was investigated by re-docking the cocrystallized ligands back to their respective receptors using Glide standard precision (SP)^59^. RMSD calculations were made using the superposition tool in Maestro, in which the docked pose was superimposed onto the crystal structure ligand conformation using the substructure recognition SMARTS.

### Virtual screening

The virtual screens were conducted using the library of drug-like decoys from Schrödinger (www.schrödinger.com) enriched with 30 known PPARγ partial agonists. The docking site was defined as a 30 × 30 × 30 Å box centered on the average of coordinates of the native ligand present in the PPARγ crystal structures. Each compound in the database was docked into the six crystal structures with one pose per ligand collected. These compounds were ranked and scored using GlideScore. For each ligand, only the conformation with the best score (lowest GlideScore) was retained for the enrichment studies.

ROC curves^61^ and enrichment factors (EF)^62^ were calculated to compare the performance of each crystal structure. ROC Enrichment metrics were calculated by using the script enrichment.py available in Maestro. Results of the virtual screening and re-docking experiments are summarized in Table S3.

Among the six crystal structures evaluated, the structure 3B3K clearly gave the best results, identifying the most active compounds with a ROC value of 0.80 and an enrichment factor of 9.5 at 2%. Furthermore, the RMSD value between the native and the re-docked pose was only 0.5 Å. Supplementary Figure S7 shows the percent of known actives found (Y axis) *vs* percent of the ranked database screened (X axis) for PDB entry 3B3K. Hence, we selected 3B3K as the most suitable structure for docking studies.

### Covalent docking

The core structure of compound **1** was retrieved from the Cambridge Structural Database (refcode: YALXIU) and modified with the fragment dictionary of Maestro. The ligand was then preprocessed with LigPrep 3.3 and optimized by Macromodel 10.7, using the MMFFs force field with the steepest descent (1000 steps) followed by truncated Newton conjugate gradient (500 steps) methods. Partial atomic charges were computed using the OPLS-AA force field.

Docking of compound **1** to PPARγ was performed with the Schrödinger CovDock algorithm, which uses both Glide and Prime^59^,^60^,^63^,^64^. It is developed to mimic the covalent ligand binding by first positioning the prereaction form of the ligand in the binding site close to the receptor reactive residue using Glide docking with positional constraints and only then generating the covalent attachment. In the prereaction docking step, the reactive residue is mutated to alanine to enable a closer approach by the reactive group of the ligand. The resultant protein-ligand geometries are ranked based on VSGB 2.0, an all-atom energy function based on OPLS force field and Generalized Born solvation model. As input, we specified Michael’s addition as the type of reaction by which the ligand binds to the receptor. The reaction is predefined to recognize the ligand reactive group with the encoded SMARTS pattern (in this case the α,β-unsaturated carbonyl moiety present in **1**), and to perform the postreaction changes in hybridization of the ligand. In the receptor section, the sulfhydryl group C285 side chain of PPARγ was selected as the reactive residue. Five output poses were generated. The top one pose, based on its Prime energy property, was selected and is presented in Fig. 4. The reaction generated a chiral center at position 16 in the (*R*)-configuration.

### Plasmids and transient transfection experiments

HEK293 cells that stably express an exogenous Flag-tagged PPARγ from a transfected PCDNA-3 vector carrying a complete PPARγ cDNA were used for transfection experiments. The PPRE-Luc plasmid contains a luciferase reporter gene under the transcriptional control of three copies of the PPRE (Peroxisome Proliferator Response Element) derived from Acyl-CoA oxidase gene fused upstream to the herpes simplex thymidine kinase (TK) promoter, as described^46^. The RSV-βGal plasmid, expressing β-galactosidase gene, driven by the strong Rous Sarcoma Virus (RSV) promoter/cassette was used as an internal control for transfection efficiency. Transient transfection assays were carried out as described in ref. 44. The same protocol was used in cotrasfection experiments with FLAG-PPARγ wt and its mutated form in which an Alanine replaces a Cysteine at position 285 (C285A). In this case, the experiments were carried out into basal HEK293T cells and the RSV-β-Gal plasmid was used as control for transfection efficiency. Similar transfection conditions were used for the treatment with GW9662. The wild type and mutant PPARγ containing expression vectors were kindly provided by Dott. Takuma Shiraki. At least three independent experiments were performed for each transfection carried out in duplicate. Luciferase activity was normalized to β-galactosidase activity and reported as fold-induction.

### Western blotting analysis

Treated and untreated cells were lysed in Ripa buffer (150 mM NaCl, 50 mM Tris-HCl, pH 7.6, 10 mM EDTA, 1% NP-40) containing also a protease inhibitors cocktail and then centrifugated at 17,000 RCF for 10 min, at 4 °C. Supernatant containing total proteins was quantified and 80 μg of each sample were separated on 12% SDS-PAGE. Western blotting assays were carried out] Protein extracts from treated and untreated cells were obtained, quantified and analyzed by Western blot as reported^46^.

### Statistical analysis of the *in vitro* assays

All experiments were performed in duplicate or triplicate with three biological replicates. Data from viability, Western blotting and transient transfection experiments were expressed as means ± SD. Data between two groups were assessed using the Student’s t test. P-values less than 0.05 were considered significant. Asterisks reported in the figures show significance degrees, set to *p ≤ 0.05, **p ≤ 0.01 and ***p ≤ 0.001.

## Additional Information

**How to cite this article**: Vasaturo, M. *et al*. A compound-based proteomic approach discloses 15-ketoatractyligenin methyl ester as a new PPARγ partial agonist with anti-proliferative ability. *Sci. Rep.* **7**, 41273; doi: 10.1038/srep41273 (2017).

**Publisher's note:** Springer Nature remains neutral with regard to jurisdictional claims in published maps and institutional affiliations.

## Supplementary Material

Supplementary Information

## Figures and Tables

**Table 1:** Thermodynamic and kinetic constants measured by SPR for compounds **1**–**9** and Rosiglitazone injected on immobilized PPARγ.

Compound	K_D_ (nM)	k_off_ (s^−1^)
**1**	25.8 ± 5.5	0.0015
**2**	1856 ± 78	0.051
**3**	4587 ± 206	0.096
**4**	No binding	
**5**	No binding	
**6**	No binding	
**7**	No binding	
**8**	No binding	
**9**	No binding	
Rosiglitazone	320.5 ± 9.7	0.0286
