# Supplementary material for: A compound-based proteomic approach discloses 15-ketoatractyligenin methyl ester as a new PPARγ partial agonist with anti-proliferative ability
Source: Sci Rep. 2017 Jan 24;7:41273. doi: 10.1038/srep41273 (PMC5259791; doi:10.1038/srep41273)
Supplement: Supplementary Information [file srep41273-s1.pdf]

Supplementary Information

A compound-based proteomic approach discloses 15-ketoatractyligenin methyl ester as a new PPARγ partial agonist with anti-proliferative ability

Michele Vasaturo[1], Lorenzo Fiengo[1,6], Nunziatina De Tommasi[1], Lina Sabatino[2], Pamela Ziccardi[2], Vittorio Colantuoni[2] Maurizio Bruno[3], Carmen Cerchia,[4] Ettore Novellino[4], Angelo Lupo[2*], Antonio Lavecchia[4*], Fabrizio Dal Piaz[1,5]

[1]Department of Pharmacy, University of Salerno, Via Giovanni Paolo II, 132, 84084 Fisciano, Italy

[2]Department of Sciences and Technologies, University of Sannio, Via port'Arsa, 11 82100 Benevento, Italy

[3]Department of Organic Chemistry, University of Palermo, Viale delle Scienze - Parco d'Orleans II, 90128 - Palermo, Italy

[4]Department of Pharmacy, "Drug Discovery" Laboratory, University of Napoli "Federico II", Via D. Montesano, 49, 80131 Napoli, Italy

[5]School of Medicine and Surgery, University of Salerno, via Salvatore Allende, 18, Baronissi (SA)

[6]PhD Program in Drug Discovery and Development, University of Salerno, Via Giovanni Paolo II 132, I-84084 Fisciano (SA), Italy

*Correspondence: lupo@unisannio.it (A.L.), antonio.lavecchia@unina.it (A.L.)

Supplemental Figures and Tables

Figure S1. Chemical modification of compound **1** for chemical proteomics experiments.

Figure S2. Western blot analysis of proteins eluted in the chemical proteomics experiments.

Figure S3. SPR sensorgrams obtained for compounds **2-8** and rosiglitazone.

Figure S4. SPR sensorgrams obtained injecting **1** on PPARγ previously incubated with the same compound.

Figure S5. PPARγ transactivation activity of compound 1 in the presence of Tween 20.

Figure S6. Structures of known PPARγ partial agonists selected as the active compound set.

Figure S7. ROC curve for the virtual screening performed on PDB entry 3B3K.

Table S1. MS analysis of peptide fragments generated in the trypsin digestion of the **1**/PPARγ LBD complex.

Table S2. MS analysis data of Hsp60 incubated with **1** and digested with trypsin.

Table S3. Cognate docking and virtual screening results for six PPARγ crystal structures.

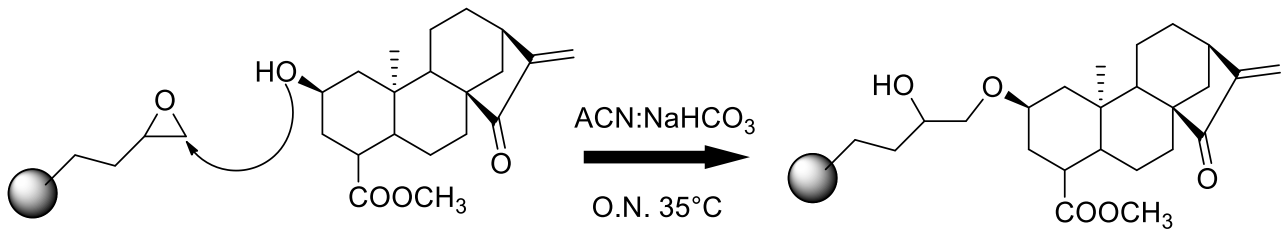

Figure S1. Chemical modification of compound **1** aimed to its immobilization for chemical proteomics experiments. Reaction conditions were optimized to preserve compound **1** biological activity.

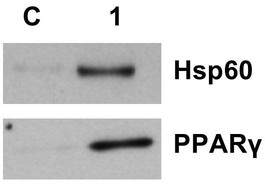

Figure S2. Western blot validation of chemical-proteomics results. Proteins eluted from control (C) and **1**-modified (**1**) beads were revealed using anti-Hsp60 and anti-PPARγ antibodies. Blots are representative of three separate experiments with similar results.

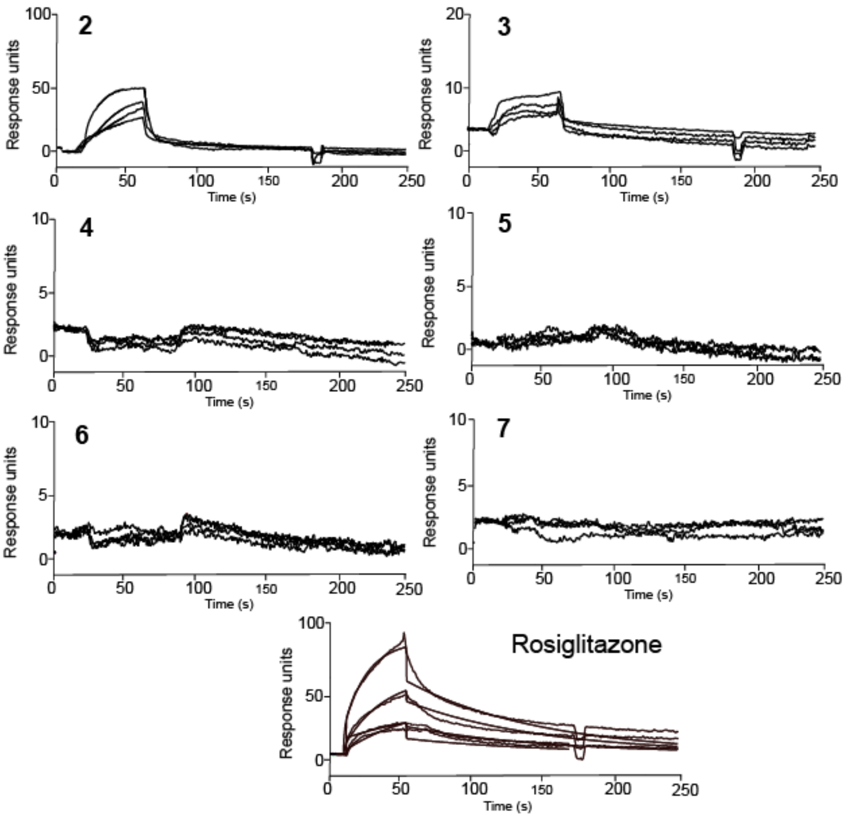

Figure S3. SPR sensorgrams achieved injecting different concentrations (from 0.025 to 1 μM) of compound **2**-**7** and of the positive control rosiglitazone on immobilized PPARγ LBD.

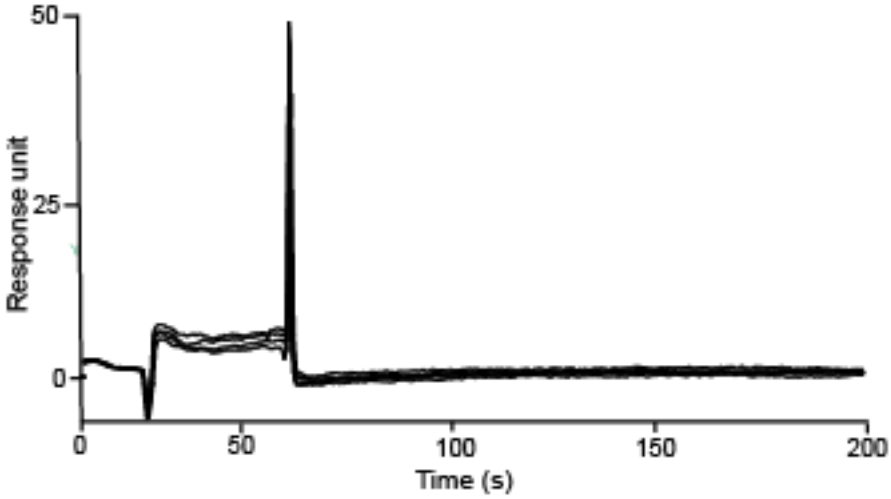

Figure S4. SPR sensorgrams obtained injecting **1** on PPARγ previously incubated with the same compound.

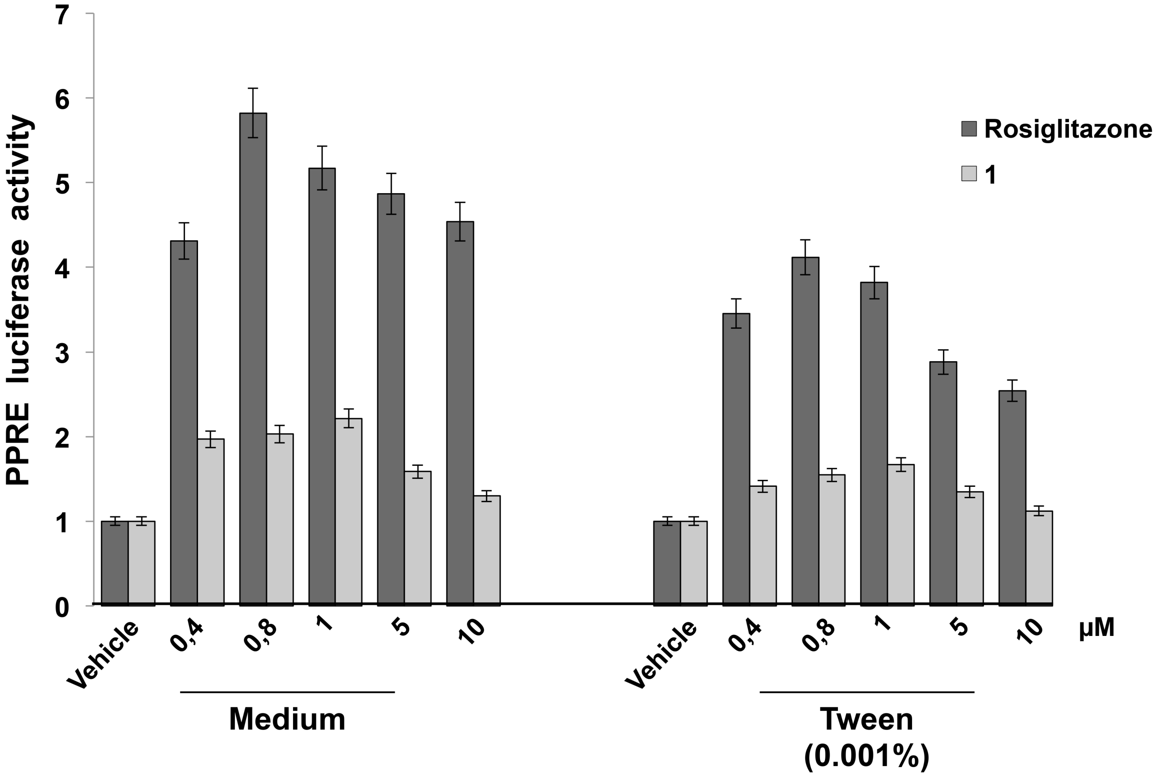

Figure S5: **PPARγ transactivation activity of compound 1 in presence of Tween 20.**

Human HEK293 cells stably expressing an exogenous Flag-tagged wild type PPARγ1 were transiently transfected with the PPRE-luciferase reporter gene and treated for 24 hs with rosiglitazone or compound 1, respectively, at the indicated doses in presence or absence of a detergent such as Tween 20 at 0.001% in the medium. Luciferase activity is reported as fold-induction after normalization to β-galactosidase activity used as control for transfection efficiency. The results are further normalized to those from cells treated with vehicle only (e. g. DMSO in the absence of compound 1 or Rosiglitazone). Data are the mean ± SD of three independent experiments performed in duplicate.

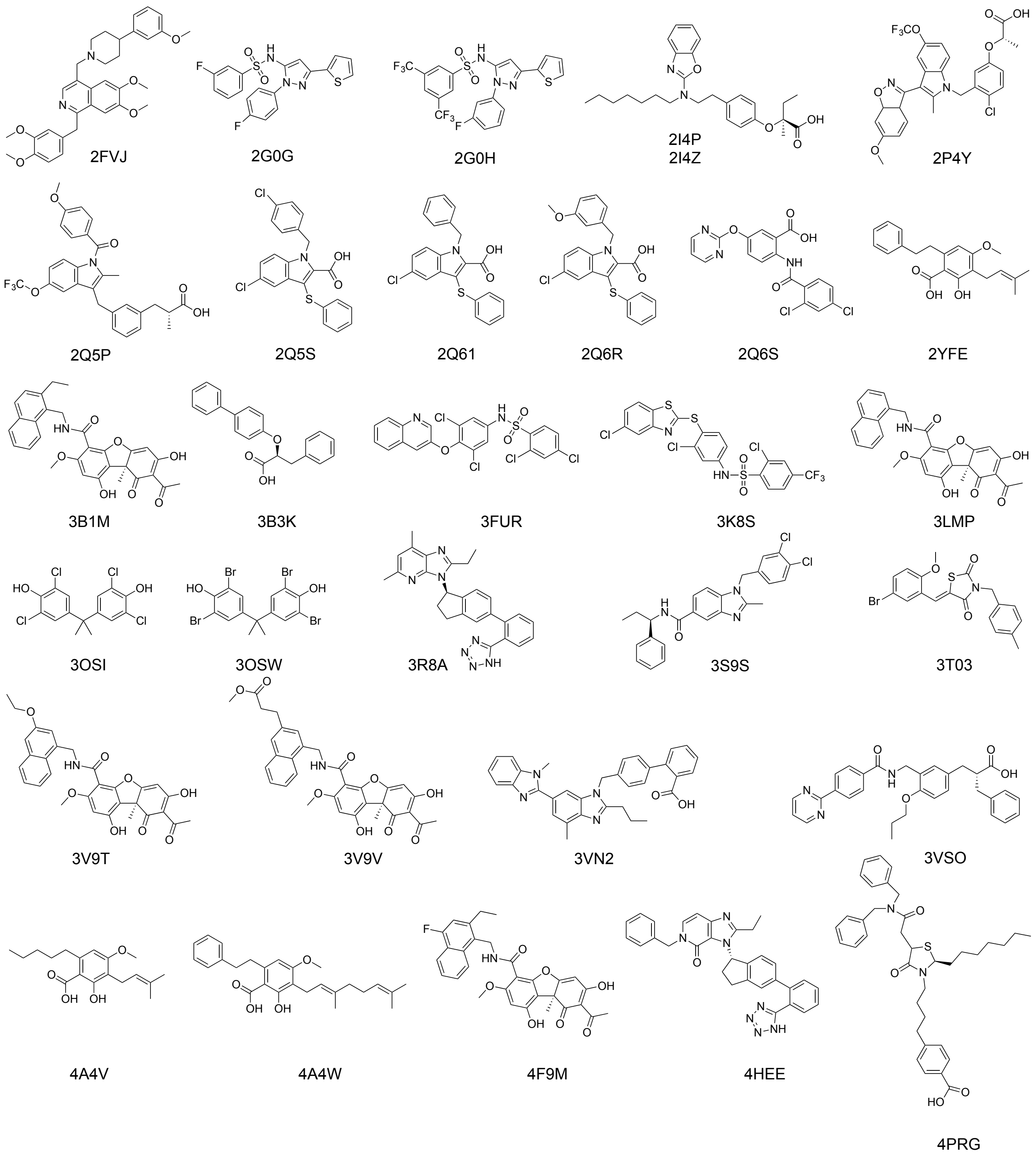

Figure S6. Structures of known PPARγ partial agonists selected as the active compound set.

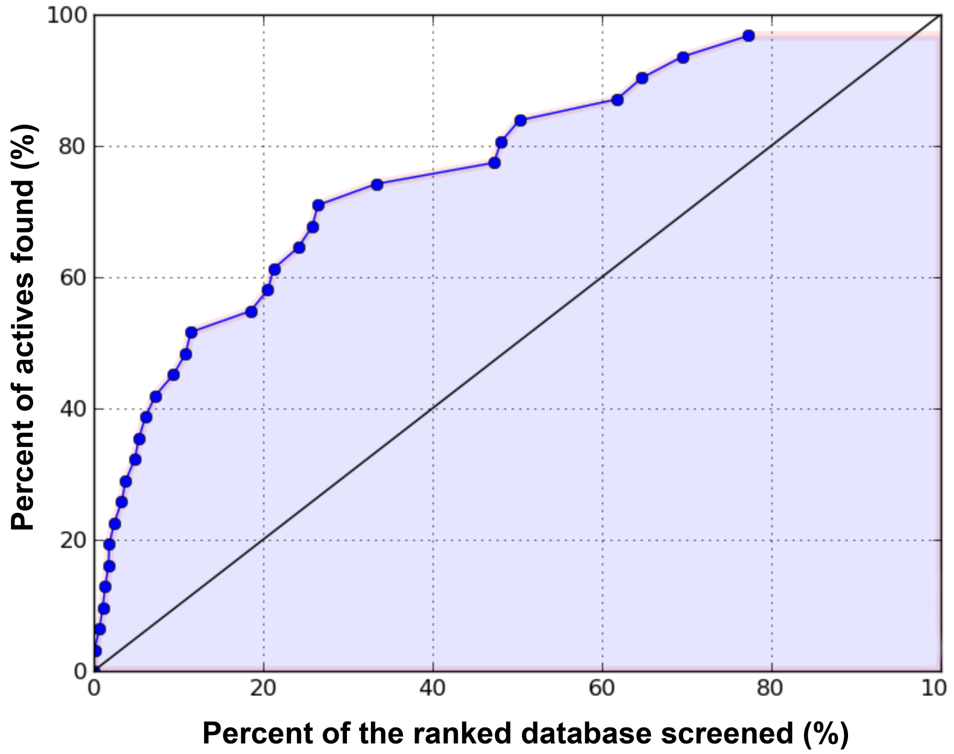

Figure S7. ROC curve for the virtual screening performed on PDB entry 3B3K.

Table S1. Mass spectrometry analysis data of the PPARγ LBD/**1** digested with trypsin.

Observed m/z	Measured MW	Peptide[1]	Theoretical MW
519.766 $[M+2H]^{2+}$	1037.516	217-224	1037.518
692.397 $[M+H]^{+}$	691.389	225-230	691.390
602.387 $[M+H]^{+}$	601.379	235-240	601.380
464.236 $[M+H]^{+}$	463.228	241-244	463.228
988.943 $[M+2H]^{2+}$	1975.871	245-261	1975.873
590.818 $[M+2H]^{2+}$	1179.621	266-275	1179.625
587.353 $[M+H]^{+}$	586.345	276-280	586.344
998.486 $[M+H]^{+}$	997.478	281-288	997.480
665.844 $[M+2H]^{2+}$	1329.673	(281-288)-**1**	1329.677
733.871 $[M+2H]^{2+}$	1465.726	289-301	1465.730
993.548 $[M+2H]^{2+}$	1985.080	302-319	1985.083
992.004 $[M+2H]^{2+}$	1981.993	320-336	1982.000
755.370 $[M+2H]^{2+}$	1508.725	337-350	1508.729
598.293 $[M+2H]^{2+}$	1194.570	358-367	1194.574
740.393 $[M+H]^{+}$	739.385	368-373	739.390
1354.734 $[M+4H]^{4+}$	5414.906	374-422	5414.959
685.853 $[M+2H]^{2+}$	1369.691	423-434	1369.699
501.339 $[M+H]^{+}$	500.331	435-438	500.332
635.316 $[M+H]^{+}$	634.308	439-443	634.311
824.490 $[M+2H]^{2+}$	1646.964	444-457	1646.972
959.480 $[M+2H]^{2+}$	1916.945	459-474	1916.955

[1] Numbers correspond to those of the first and the last AA in the human PPARγ (isoform 1) sequence.

Table S2. Mass spectrometry analysis data of Hsp60 incubated with **1** and digested with trypsin.

Observed m/z	Measured MW	Peptide[1]	Theoretical MW
1057,079 $[M+2H]^{2+}$	2112,138	38-58	2112,132
672,865 $[M+2H]^{2+}$	1343,710	61-72	1343,708
854,0884 $[M+3H]^{3+}$	2559,251	97-121	2559,241
855,473 $[M+H]^{+}$	854,465	134-141	854,461
795,418 $[M+3H]^{3+}$	2383,232	158-180	2383,223
752,886 $[M+2H]^{2+}$	1503,755	206-218	1503,749
695,358 $[M+2H]^{2+}$	1388,672	222-233	1388,698
772,877 $[M+2H]^{2+}$	1543,733	237-249	1543,723
960,044 $[M+2H]^{2+}$	1918,069	251-268	1918,064
1183,174 $[M+2H]^{2+}$	2364,339	269-290	2364,326
912,595 $[M+2H]^{2+}$	911,589	293-301	911,580
1033,179 $[M+3H]^{3+}$	3096,521	315-344	3096,507
844,517 $[M+H]^{+}$	843,509	345-352	843,506
1019,519 $[M+2H]^{2+}$	2037,027	371-387	2037,015
901,540 $[M+H]^{+}$	900,532	397-405	900,528
617,305 $[M+2H]^{2+}$	1232,596	406-417	1232,588
960,517 $[M+H]^{+}$	959,509	421-429	959,504
814,448 $[M+2H]^{2+}$	1626,888	430-446	1626,876
857,921 $[M+2H]^{2+}$	1713,831	447-462	1713,824
608,338 $[M+2H]^{2+}$	1214,665	482-493	1214,651
836,710 $[M+3H]^{3+}$	2507,120	494-516	2507,102
837,816 $[M+2H]^{2+}$	1673,621	555-573	1673,613

[1]: Numbers correspond to the number of the first and the last AA in human Hsp60 sequence

Table S3. Cognate docking and virtual screening results for six PPARγ crystal structures.

PDB	Resolution (Å)	Cognate Ligand RMSD (Å)	ROC	$EF^{2\%\ a}$	$EF^{5\%\ b}$	$EF^{10\%\ c}$
2G0G	2.54	0.61	0.65	7.9	3.8	3.2
2G0H	2.3	0.53	0.58	4.8	2.6	1.9
2I4P	2.1	5.9	0.77	9.5	5.8	3.9
2I4Z	2.25	8.3	0.71	4.8	5.1	2.9
3B3K	2.6	0.5	0.80	9.5	6.4	4.5
4PRG	2.9	10.6	0.71	4.8	3.2	3.6

[a]Maximum enrichment factor at 2 % = 22.7.
[b]Maximum enrichment factor at 5 % = 19.7.
[c]Maximum enrichment factor at 10 % = 10.0.
